# A major role for Nrf2 transcription factors in cell transformation by KSHV encoded oncogenes

**DOI:** 10.3389/fonc.2022.890825

**Published:** 2022-09-21

**Authors:** Daiana Sapochnik, Ana R. Raimondi, Victoria Medina, Julian Naipauer, Enrique A. Mesri, Omar Coso

**Affiliations:** ^1^ CONICET-Universidad de Buenos Aires, Instituto de Fisiología, Biología Molecular y Neurociencias (IFIBYNE), Buenos Aires, Argentina; ^2^ Departamento de Fisiología, Biología Molecular y Celular, Universidad de Buenos Aires, Facultad de Ciencias Exactas y Naturales, Buenos Aires, Argentina; ^3^ University of Miami- Center for AIDS Research (UM-CFAR)/Sylvester Comprehensive Cancer Center (CCC) Argentina Consortium for Research and Training in Virally Induced AIDS-Malignancies, University of Miami Miller School of Medicine, Miami, FL, United States; ^4^ Viral Oncology Program, Sylvester Comprehensive Cancer Center, Miami Center for AIDS Research, Department of Microbiology & Immunology, University of Miami, Miami, FL, United States

**Keywords:** Nrf2, HO-1 (heme oxygenase-1), gene expression regulation, KSHV-vGPCR, signal transduction

## Abstract

Kaposi’s sarcoma (KS) is the most common tumor in AIDS patients. The highly vascularized patient’s skin lesions are composed of cells derived from the endothelial tissue transformed by the KSHV virus. Heme oxygenase-1 (HO-1) is an enzyme upregulated by the Kaposi´s sarcoma-associated herpesvirus (KSHV) and highly expressed in human Kaposi Sarcoma (KS) lesions. The oncogenic G protein-coupled receptor (KSHV-GPCR or vGPCR) is expressed by the viral genome in infected cells. It is involved in KS development, HO-1 expression, and vascular endothelial growth factor (VEGF) expression. vGPCR induces HO-1 expression and HO-1 dependent transformation through the Ga13 subunit of heterotrimeric G proteins and the small GTPase RhoA. We have found several lines of evidence supporting a role for Nrf2 transcription factors and family members in the vGPCR-Ga13-RhoA signaling pathway that converges on the HO-1 gene promoter. Our current information assigns a major role to ERK1/2MAPK pathways as intermediates in signaling from vGPCR to Nrf2, influencing Nrf2 translocation to the cell nucleus, Nrf2 transactivation activity, and consequently HO-1 expression. Experiments in nude mice show that the tumorigenic effect of vGPCR is dependent on Nrf2. In the context of a complete KSHV genome, we show that the lack of vGPCR increased cytoplasmic localization of Nrf2 correlated with a downregulation of HO-1 expression. Moreover, we also found an increase in phospho-Nrf2 nuclear localization in mouse KS-like KSHV (positive) tumors compared to KSHV (negative) mouse KS-like tumors. Our data highlights the fundamental role of Nrf2 linking vGPCR signaling to the HO-1 promoter, acting upon not only HO-1 gene expression regulation but also in the tumorigenesis induced by vGPCR. Overall, these data pinpoint this transcription factor or its associated proteins as putative pharmacological or therapeutic targets in KS.

## Introduction

Exposure of cells to environmental toxicants and potential carcinogens has been linked to pathologic processes, including neurodegenerative and cardiovascular diseases, as well as to cancers (1). Eukaryotic cells have developed complex responses to detoxify potentially harmful substances and maintain cellular redox homeostasis in which different signaling cascades participate. The response involves the induction of cytoprotective and detoxifying enzymes consisting of phase I (cytochrome P450s) and phase II (detoxifying and antioxidant proteins) enzymes (2). The expression of these genes attempts to restore the cell to a basal state, preventing damage to cellular components sensitive to redox changes (i.e., proteins, lipids, and DNA) (3). One such enzyme is Heme oxygenase-1 (HO-1), an inducible and ubiquitous 32-kDa enzyme that regulates heme metabolism and iron levels by catalyzing the degradation of the heme group. The products of this enzymatic reaction are carbon monoxide, free iron, and biliverdin. This final product is subsequently reduced to the antioxidant bilirubin (4). HO-1 activity can be regulated at different levels, but it depends primarily on the control of HO-1 expression at the transcriptional level (4–6). A variety of stress-inducing stimuli, antioxidants, growth factors, and hormones can induce HO-1 expression (7–10). HO-1 has been considered a cytoprotective molecule because of heme metabolism products’ antioxidant properties. It has been involved in several physiological responses against oxidative and cellular stress and inflammation (5). However, several studies have now expanded this notion and defined HO-1 as an important regulator of the physiology of the vasculature, vascular endothelial growth factor (VEGF) secretion, endothelial cell cycle control, proliferation, angiogenesis, and tumorigenesis (6, 11–14).

The transcriptional regulation of HO-1 is mainly controlled by Nrf2, a transcription factor of the leucine zipper-type, a cap’n’collar bZip protein ubiquitously expressed and responsible for the basal and inducible expression of proteins involved in the oxidative stress response, drug metabolism, cytoprotection, apoptosis, differentiation, proliferation, and growth (15). Nrf2 undergoes spatial-temporal regulation switched on and off by tightly regulated mechanisms. Proteasomal degradation regulates the cell’s response to inflammatory, hypoxic, oxidative, and xenobiotic stimuli. In unstressed conditions or resting cells, the level of Nrf2 protein is maintained at very low levels by its inhibitor Keap1 which sequesters Nrf2 in the cytosol and facilitates its degradation *via* the proteasome. Several mechanisms by which Nrf2 can be regulated independently of Keap1 were described (16, 17). In the presence of Nrf2 inducers, Nrf2 is liberated from Keap, translocates to the nucleus, and forms a heterodimer with small masculoaponeurotic fibrosarcoma (Maf) binding to the Antioxidant Response Element (ARE), a cis-acting enhancer sequence (TCAG/CXXXGC) (15, 18, 19). The genes that are Nrf2-regulated can be classified into phase II enzymes, antioxidants, molecular chaperones, DNA repair enzymes, and anti-inflammatory response proteins (20). Importantly, the Nrf2 promoter displays an ARE sequence within its promoter region to initiate its transcription further enhancing the adaptive cell defense response (15). Nrf2 seems to play a dual role in cancer, potentially acting as both a tumor suppressor and an oncogenic factor. Raised Nrf2 levels have been detected in an array of cancer tissues including lung (21, 22) and pancreas (23, 24). It is proposed that this provides cells with enhanced chemo-resistance as well as supports increased proliferation thus promoting cancer growth and development (23). Nrf2-deficient mice are more susceptible to toxicity by compounds such as paracetamol and tobacco smoke and too many diseases. Interestingly, Nrf2 ^-^/^-^ mice do survive and can procreate which suggests that Nrf2 is not necessarily vital for survival in unstressed cells and is only called upon in the presence of stress or insult (25). However, Nrf2 plays a major role in health and disease and therefore is a potential therapeutic target. This fact highlights the need to understand and determine whether gene expression regulation of Nrf2 would be beneficial in both the short and long-term and its intermediate signaling mediators.

The most frequent type of tumor in AIDS patients is Kaposi sarcoma (KS), formed by spindle cells derived from endothelial cells transformed by KSHV (26, 27). The product of orf 74 in the KSHV genome is a constitutively active G protein-coupled receptor (vGPCR) that plays an important role in the development of KSHV-induced oncogenesis (26, 28–31). Only a few cells in KS-like lesions express vGPCR (28), however, down-regulation of the receptor in these cells results in a decreased expression of angiogenic factors and tumor regression (32, 33). vGPCR is homologous to the mammalian interleukin-8 receptor. A mutation confers vGPCR its constitutive, ligand-independent activity (34–36). It has been shown that the expression of vGPCR in fibroblasts induces transformation, angiogenesis in endothelial cells (EC), and angioproliferative KS-like lesions in mice (28, 31, 37). HO-1 expression is induced during KSHV infection of human dermal microvascular endothelial cells (DMVEC) and is highly expressed in biopsy tissues from oral AIDS-Kaposi sarcoma lesions (38). Moreover, KSHV induction of HO-1 in lymphatic EC (LEC) occurs in two distinct phases, a transient phase upon acute infection and a sustained phase coincident with the establishment of viral latency (39). Previous work from our laboratory and collaborators shows that vGPCR induces HO-1 mRNA and protein levels in fibroblasts and endothelial cells and that these facts correlate with increased cell proliferation, survival, and VEGF-A expression, one of the determinant events in KS development. Inhibition of HO-1 expression or activity impairs the tumorigenesis induced by vGPCR in allograft tumor animal models (40). Several studies show that vGPCR contributes to KS development by switching on a complex network of signaling pathways (29, 41). vGPCR activates downstream effectors by coupling to different subunits of heterotrimeric G proteins (42–44). We have shown that the expression of both Ga12 and Ga13 subunits mimicked vGPCR-induced HO-1 expression and transformation through the small GTPase RhoA. Reduced expression of RhoA impairs vGPCR-induced VEGF expression and secretion, cell survival and proliferation, and transformation both in cell culture and in a murine allograft tumor model (40, 45). Despite the implication of HO-1 as a vGPCR downstream target, the nature of the molecular pathways connecting the receptor to HO-1 expression regulation remains unknown.

Previous reports have shown that KSHV *de novo* infection of HMVEC-d requires ROS for Nrf2 activation during the early stages of infection and establishment of latency and observed activated Nrf2 levels in KSHV positive KS and Primary Effusion Lymphoma (PEL) lesion cells (46). Moreover, two simultaneous Nrf2 activation pathways necessary for the sustained expression of the Nrf2 target gene have been shown to occur in KSHV-infected long-term-infected telomerase-immortalized endothelial (TIVE-LTC) cells (47).

In this study, using a combination of biological models that include cells transformed by vGPCR in culture, tumors in mice, and KSHV full genome-bearing cells, including KSHV-Bac16 based mutant system with vGPCR deletion, we have shown that the effect of vGPCR signaling on HO-1 expression and tumorigenesis is mediated by ARE sites and its associated transcription factor Nrf2. Moreover, vGPCR not only affects the transcriptional activation of Nrf2 but also induces Nrf2 nuclear translocation. Nrf2 transcriptional activity and nuclear translocation are mainly mediated by vGPCR-induced activation of the Gα12/13-RhoA-ERK1/2 signaling pathway.

## Experimental procedures

### DNA constructs

The plasmid pHO-1-Luc was provided by J. Alam and contained a 15- kb murine HO-1 promoter upstream of a luciferase gene (reporter gene vector pSK-luc) as described previously (48). pCEFL-AU5-vGPCR was constructed in Dr. Gutkind’s laboratory introducing vGPCR cDNA as a Bgl2/Not1 fragment in pCEFLAU5 (28). The reporter construct pGL3_Nrf2-3xARE-Luc (minimum promoter containing three ARE sites in tandem, T/CGCTGAGTCA) was a gift of M. Marinissen, and the pG5 Luc (minimum promoter containing 5 sites for GAL4 binding) was purchased from Promega. The expression plasmid for Nrf2 WT, pCDNA3 His B – V5 Nrf2 (Wild Type), was a kind gift from M. McMahon y J. Hayes and previously described (49). Briefly, the PCR amplification of the murine Nrf2 coding sequence and 50 nucleotides of the 5′-untranslated region was ligated into EcoRV-digested pcDNA3.1/V5HisB (Invitrogen), allowing expression of mNrf2-V5-his fusion protein from a CMV promoter. The expression plasmid for Nrf2 Dominant Negative (Nrf2 DN) (reporter gene vector pEF) was provided by J. Alam and described previously (48). The expression plasmids pCDNA3_Gα12-QL and pCEFL_HA_Gα13-QL were obtained by cloning the constitutively active forms of both cDNAs and cloned as Bgl2/Not1 fragments into pCDNA3 and pCEFL-HA, respectively. pCEFL_AU5_RhoA-QL and pCEFL_Rho-N19 Dominant Negative were cloned as BamH1/EcoR1 fragments and introduced in the respective vectors in Dr. Gutkind’s laboratory who provided them as a gift (40, 50, 51). The expression vectors for the MEKs were previously described (52).

### Cell lines and transfections

NIH3T3 fibroblasts were received from Dr. S. Gutkind’s laboratory and maintained in Dulbecco’s modified Eagle’s medium (DMEM) (Invitrogen) supplemented with 10% calf serum. Stable transfections of NIH3T3 for vGPCR were received from Dr. S. Gutkind’s laboratory and described previously (40). Stable transfections of NIH3T3_vGPCR for different shRNAs were performed using the Lipofectamine Plus Reagent (Invitrogen). NIH3T3_vGPCR cells were plated at 60% confluence in 10 cm plates and transfected with 2ug of shRNA (shScramble and shRNAs targeted against Nrf2 - GIPZ Nfe2l2 shRNA Thermo RMM4532-EG18024). Transfected cells were selected with 750 ug/ml G418 (Promega Corp., Madrid, Spain) and 0.4ug/ml Puromycin (Invitrogen). mECs were obtained from Balb/C mice (NCI, Bethesda, MD) as previously described (33). By transfection with KSHVBac36, the vector containing the insert with the genome of KSHV in Bacterial Artificial Chromosome (KSHVBac36), mECK36 cells were generated. mECKnull is a variant of the latter that lost the plasmid and was subsequently used as the source for the generation of mECK16-delta-Revertant or mECK16-delta–vGPCR cell lines, which express reinstalled KSHV genomes in its complete or devoid of vGPCR versions, respectively (53).

### Luciferase reporter assays

Cells were transfected with different expression plasmids together with 1ug of the indicated reporter plasmid per well in 6-well plates. In all cases, the total amount of plasmid DNA was adjusted with pcDNA3 empty and 0.2ug of pCDNA3-b- galactosidase. *Firefly* luciferase activity in cellular lysates was assayed using the luciferase reporter system catalog number E1500 (Promega Corp.), and light emission was quantified using a luminometer (Junior Berlthold). To study transcriptional activation domains of transcription factors, we used the Luciferase GAL4 reporter system, where a Gal4 DBD – Nrf2 TAD vector expresses a fusion protein containing the DNA binding domain of GAL4 and the transactivation domain of Nrf2, is co-transfected with the pG5 Luc. When co-transfected in the same cell, these two constructs allow for the assessment of the regulation of gene expression by signal transduction pathways that impinge on the transcription factor Nrf2 using luciferase as a reporter gene. All assays were performed in three biological replicates and measured in triplicates for quantification.

### RT-qPCR

Total cellular RNA was isolated using TRIReagent (Genbiotech-MRC) using the manufacturer’s protocols. RNA concentration was measured by Nanodrop™ (Thermo Fisher), and 2ug of RNA was treated with RNase-free DNase I (Invitrogen). DNase treatment was performed for 15 min at room temperature, followed by EDTA treatment for 10 minutes at 65 ° to stop the DNase. After DNase treatment, the samples were incubated for 5 minutes at 65 ° in the presence of 500ng OligodT (Genbiotech). cDNA synthesis was performed using MMLV (Moloney murine leukemia virus) reverse transcriptase (RT) (Promega) with RNaseOUT (Invitrogen) for 50 minutes at 37°. qPCR was performed using 1ul of cDNA in a BioRad CFX96 instrument with 9ul of the Master Mix qPCR 2.0 (PB-L, Productos Bio-Logicos), including SYBR Green detection. qPCR cycles: STEP 1 95° 3min; STEP 2 (x40) 95° 20seg, 62° 20 seg and 72° 30 seg with reading; STEP 3 95° 1 min; STEP 4 Melting curve 60° 30seg, 95° 30seg (temperature of the sample is then increased incrementally as the instrument continues to measure fluorescence). Luciferase mRNA was quantified by qPCR using the following primer set: forward (5-CCGCCGTTGTTGTTTTG- 3) and reverse (5-ACACAACTCCTCCGCGC-3). For vGPCR, the primer sequences were: forward (5-AGGAGCGATAGATATACTG-3) and reverse (5-CAACACTTCTGCCAATAG-3). Luciferase and vGPCR mRNA expression were normalized against the Beta-galactosidase mRNA, in which primer sequences were: forward (5-CCACGGAGAATCCGACG-3) and reverse (5-GCGAGGCGGTTTTCTCC-3). In every run, melting curve analysis was performed to verify the specificity of products and water and No–RT controls. Three biological replicates were used for each sample and measured in triplicates in the qPCR analysis. Data were analyzed using the ΔΔCT method previously described (54); we used this method because the genes were amplified with comparable efficiencies. Target gene expression was normalized to Beta-galactosidase by taking the difference between CT values for target genes and Beta-galactosidase (ΔCT value). These values were then calibrated to the control sample to give the ΔΔCT value. The fold target gene expression is given by the formula: 2–ΔΔCT. We used Beta-galactosidase as a reference gene to normalize transfected cells.

### Western blot

Nrf2 and phospho-Nrf2 were detected by Western blotting with anti-Nrf2 (Abcam ab89443) and anti-phospho-Nrf2 (Abcam ab76026). For kinase analysis, we used anti-ERK2 (Santa Cruz: sc-154), Anti-Phospho–ERK1/2 (Santa Cruz sc-7383), Anti-p38 (Santa Cruz sc-535-G), Anti-Phospho-p38 (Cell Signaling 9211), Anti-JNK (Santa Cruz sc- 474-G), Anti-Phospho–JNK (Cell Signaling 9255S), Anti-Akt1 (Santa Cruz sc-1618), Anti-Phospho-Akt1/2/3 (Santa Cruz sc-16646-R), Anti-Keap1 (Cell Signaling 8047) and anti-HO-1 (Cell Signaling 43966). Proteins were visualized by enhanced chemiluminescence detection (Amersham Biosciences) using secondary antibodies coupled to horseradish peroxidase or secondary antibodies coupled to fluorophores and detected using an Odyssey System (Li-cor). The MEK inhibitor PD98059 (catalog number 513000) was purchased from Calbiochem (San Diego, California).

### Indirect immunofluorescence

NIH3T3, NIH3T3_vGPCR, NIH3T3_Gα12-QL, NIH3T3 Gα13-QL, and NIH3T3 RhoA-QL cells were seeded on glass coverslips. Cells were serum-starved for 24 h, washed twice with 1ml PBS, and then fixed and permeabilized with 4% formaldehyde and 0.05% Triton X-100 in 1ml PBS for 10 min. After washing with PBS, cells were blocked with 1% bovine serum albumin and incubated with anti Nrf2 (Abcam) as primary antibody O.N. at 4C°. Following incubation, cells were washed three times with 1ml PBS and then incubated for an additional hour with the corresponding secondary antibody (1:1000) conjugated with fluorescein isothiocyanate (Molecular Probes). Cells were washed three times with 1ml PBS and stained with Propidium Iodide (1 ug/ml) (Molecular Probes) in the last wash. Coverslips were mounted in Mowiol mounting medium (Sigma) and viewed using a confocal microscope (Fluoview FV300, Olympus, Japan). For nucleus/cytoplasm ratio quantification of protein localization, we used ImageJ and the results were displayed in the graph as fluorescence intensity ratio. Values near one indicate homogenous protein localization, while values over one indicate mainly nuclear localization, and values below one indicate localization mainly cytoplasmically. For mouse-KS tumor immunofluorescence analysis, we used frozen mouse KSHV (+) and KSHV (–) tumor samples obtained as previously described (33, 55). Briefly, mECK36 KSHV (+) cells injected subcutaneously into the flanks of nude mice form mouse KSHV (+) tumors 5 weeks after injection. KSHV (–) tumor cells injected subcutaneously into the flanks of nude mice form KSHV (–) tumors 3 weeks after injection. Immunofluorescence assay (IFA) of mECK16-deltavGPCR, mECK16Δ-Revertant, Mouse KSHV-positive, and KSHV-negative tumors was performed as previously described (56). Briefly, cells were fixed in 4% paraformaldehyde for 10 min and washed with PBS. Cells were permeabilized in 0.2% Triton-X/PBS for 20 min at 4°C. After blocking with 3% of BSA in PBS and 0.1% Tween 20 for 60 min, samples were incubated with primary antibodies overnight at 4C. After PBS washing, samples were incubated with fluorescent secondary antibodies for 1 hour (Molecular Probes), washed, and mounted with ProLong Gold antifade reagent with DAPI (Molecular Probes).

### Tumor xenografts in athymic nude mice and antitumor effect of Nrf2 silencing

NIH3T3, NIH3T3_vGPCR, NIH3T3_vGPCR_shScramble, NIH3T3_vGPCR_shNrf2 stable cell lines were used to induce tumor allografts in 7-week athymic (*nu/nu*) nude mice. Cells were harvested, washed, counted, and resuspended in PBS. 1 X 10^6^ NIH3T3_vGPCR (control) or NIH3T3_vGPCR_shNrf2 cells were injected subcutaneously in the right flank of six and five nude mice, respectively. Mice were monitored twice weekly until each animal developed one tumor in the area of the cell injection. Tumor volume and body weight were measured every other day during the investigation period. Tumor volumes (*V*) were determined by the formula *V*=Lx*W* ^2^x0.5, with *L* being the longest cross-section and *W* the shortest. Data are mean ± S.E.M expressed as tumor volume (cm^3^), calculated as described above. Animals from each group were euthanized for tissue retrieval at the various time points indicated in the study. Using standard protocols, the tissues were fixed in 4% buffered paraformaldehyde overnight, dehydrated, and embedded in paraffin. H&E-stained sections were used for diagnostic purposes.

All animal studies were carried out according to the Institutional Animal Care and Use Committee of the Facultad de Ciencias Exactas y Naturales (FCEN) the University of Buenos Aires approved protocol, and local government regulations (Servicio Nacional de Sanidad y Calidad Agroalimentaria, RS617/2002, Argentina). All mice were maintained under a 12:12 h dark/light cycle with food and water *ad libitum*. They were grouped-housed (3-5 mice per cage). Male and female mice were used and randomly assigned to the different experimental groups. All mice were euthanized by Carbon Dioxide inhalation using especially appropriate equipment of the Animal Facility.

### Statistical analysis

Sample sizes were estimated based on variance obtained from previous studies, an alpha level of 0.05, and statistical power >0.8. To minimize any possible bias during tumor measurements, mice from both experimental groups were housed together and were evaluated in random order by experimenters blinded to the treatment conditions. No exclusion criteria were *a priori* established, and all injected mice were included in the analysis. Normality of data distributions was estimated with Shapiro-Wilk tests, two-tailed parametric statistics were used in all cases, and the threshold for significance was set at p = 0.05. The *p* values are indicated along with statistical parameters, such as the number of cells and animals used in each experiment in the results and the figure legends. Statistical analysis was performed with GraphPad Prism 9 software. Group differences were analyzed by student t-test or ANOVA followed by Dunnet Test. Experimental groups are compared to the control. Two-way repeated measures ANOVA was used for tumor volume analysis. A *p-value <*0.05 (*) was considered statistically significant.

### Image analysis and quantification

Different band intensities corresponding to Western blot detection of protein samples were quantified using the ImageJ software.

## Results

### ARE elements are involved in the vGPCR-mediated activation of HO-1

**Figure 1:**
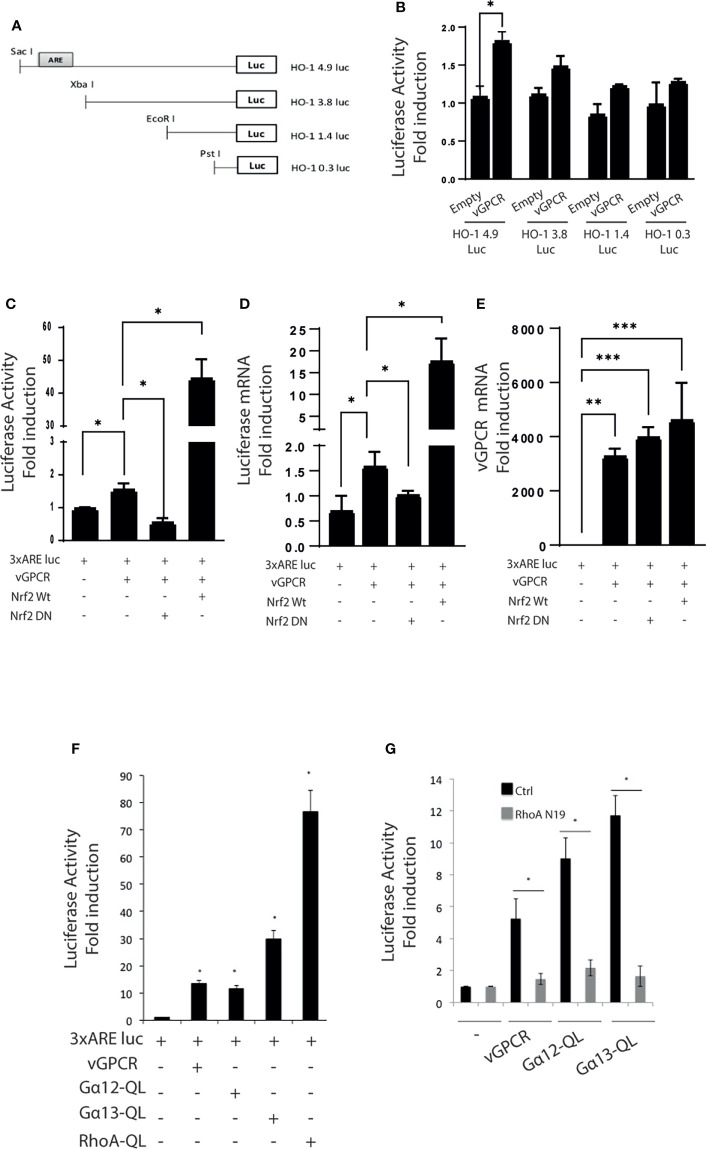
**(A)** Serial deletions of the 4.9Kb region of the HO-1 promoter. **(B)** Luciferase activity of serial deletions of the HO-1 promoter after co-transfection of the reporter construct with a plasmid that expresses vGPCR. The results are expressed as fold induction relative to cells transfected with the 4.9Kb promoter construct. **(C)** Luciferase activity of a minimum promoter with three ARE sites in tandem (3xARE Luc) after transfection of vGPCR; effect of co-transfection with vGPCR and Nrf2 WT, and effect of co-transfection with vGCPR and Nrf2 DN. The results are expressed as fold induction relative to control cells (transfected with the reporter and an empty vector). **(D)** Fold-changes of Luciferase mRNA expression were assessed by RT-qPCR in triplicate and are presented as means ± SD. **(E)** Fold-changes of vGPCR mRNA expression were assessed by RT-qPCR in triplicate and are presented as means ± SD. **(F)** Luciferase activity of the 3xARE Luc reporter constructs after transfection with plasmids expressing vGPCR, Gα12-QL, Gα13-QL, and RhoA-QL (constitutively active forms of Gα12, Gα13, and RhoA, respectively). The results are expressed as fold induction relative to control cells (transfected with the reporter and an empty vector). **(G)** Luciferase activity of the 3xARE Luc after co-transfection with vGPCR, Gα12-QL, and Gα13-QL with or without RhoA-N19 (a dominant negative form of RhoA). The results are expressed as fold induction relative to control cells (transfected with the reporter and an empty vector). *P-value <*0.05 (*). P-value <0.002 (**). P-value <0.0002 (***).

We performed Luciferase Reporter Assays to identify the elements within the HO-1 promoter responsible for vGPCR activation. For this aim, we used a reporter construct containing 4.9Kb of the HO-1 promoter (HO-1 4.9 Luc) and serial deletions (HO-1 3.8 Luc, HO-1 1.4 Luc, and HO-1 0.3 Luc) (**Figure 1A**). As shown in **Figure 1B**, the loss of the proximal ARE reduces the transcriptional activity of the HO-1 (4.9Kb) promoter. This result suggests that the ARE sequence might be responsible for HO-1 promoter activation by vGPCR. Next, we used a minimum promoter containing three ARE sites in tandem (3xARE Luc) to determine the role of the HO-1 ARE sites in activating the HO-1 promoter by vGPCR. We co-transfected this reporter with expression plasmids for Nrf2 WT and Nrf2 Dominant Negative (DN), lacking the Nrf2 transactivation domain as control of responsiveness of the reporter (**Supplementary Figure 1**). To evaluate the effect of over-expression of vGPCR, we co-transfected the 3xARE Luc reporter with expression plasmids for vGPCR. As seen in **Figure 1C**, vGPCR produced an increment in the reporter activity, indicating that vGPCR may activate ARE sites in target genes. To determine if the transcription factor Nrf2 mediated this effect, we co-transfected vGPCR with plasmids that express Nrf2 WT or Nrf2 DN. Interestingly co-transfection with vGPCR and Nrf2 WT produce a higher induction than the observed for vGPCR alone, suggesting that vGPCR and Nrf2 may be linked by a common signaling pathway. The induction produced by vGPCR was abolished by Nrf2 DN. Altogether, these results (**Figure 1C**) suggest that vGPCR activates ARE sites through Nrf2. These results were confirmed by RT-qPCR analysis of Luciferase mRNA expression (**Figure 1D**). vGPCR over-expression was confirmed by RT-qPCR (**Figure 1E**).

Like many GPCRs, vGPCR activates downstream effectors like Gα12, Gα13, and RhoA. To determine if those proteins were involved as mediators in the activation mechanism of the ARE sites by vGPCR, we performed luciferase assays with the 3xARE Luc construct co-transfected with expression vectors for vGPCR and downstream effectors. As shown in **Figure 1F**, vGPCR and its downstream effectors activate transcriptional response at the ARE sites. Moreover, the activation of ARE sites by vGPCR, Gα12, and Gα13 are RhoA dependent, as the co-transfection with RhoA Dominant Negative (RhoAN19) produces a decrease in the activation of the reporter when compared with the effect produced by vGPCR, Gα12 and Gα13 alone (**Figure 1G**). These results suggest that vGPCR, Gα12, and Gα13 activate ARE sites through RhoA.

### Key role of Nrf2 on HO-1 activation by vGPCR

**Figure 2:**
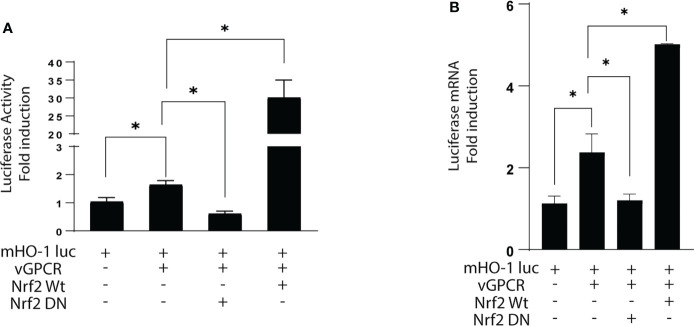
**(A)** Luciferase activity of the murine HO-1 promoter after transfection with vGPCR; co-transfection with Nrf2 WT and the dominant negative Nrf2 DN. The results are expressed as fold induction relative to control cells (transfected with the reporter and an empty vector). **(B)** Fold-changes of Luciferase mRNA expression were assessed by RT-qPCR in triplicate and are presented as means ± SD. (*P <0.05).*p-value <*0.05 (*).

To evaluate the role of Nrf2 in the induction of the HO-1 promoter by vGPCR, we performed Luciferase Reporter Assays. We used a reporter with the luciferase gene upstream of the murine HO-1 promoter. As seen in **Figure 2**, vGPCR induces an increment in the activity of the HO-1 promoter when compared to cells transfected with a control plasmid (pCDNA3.1 empty). To demonstrate that this effect was mediated through Nrf2, we co-transfected vGPCR with Nrf2 WT or with Nrf2 DN. The effect of co-transfection of vGPCR and Nrf2 WT in HO-1 promoter was bigger than the effect of vGPCR alone. Interestingly, the co-transfection of vGPCR with Nrf2 DN produced a decrease of about 30% respective to the effect of vGPCR alone. These results suggest that Nrf2 plays a key role in activating the HO-1 promoter by vGPCR (**Figure 2A**). These results were confirmed by RT-qPCR analysis of Luciferase mRNA expression (**Figure 2B**).

### vGPCR activates Nrf2 TAD and induces Nrf2 nuclear translocation

**Figure 3:**
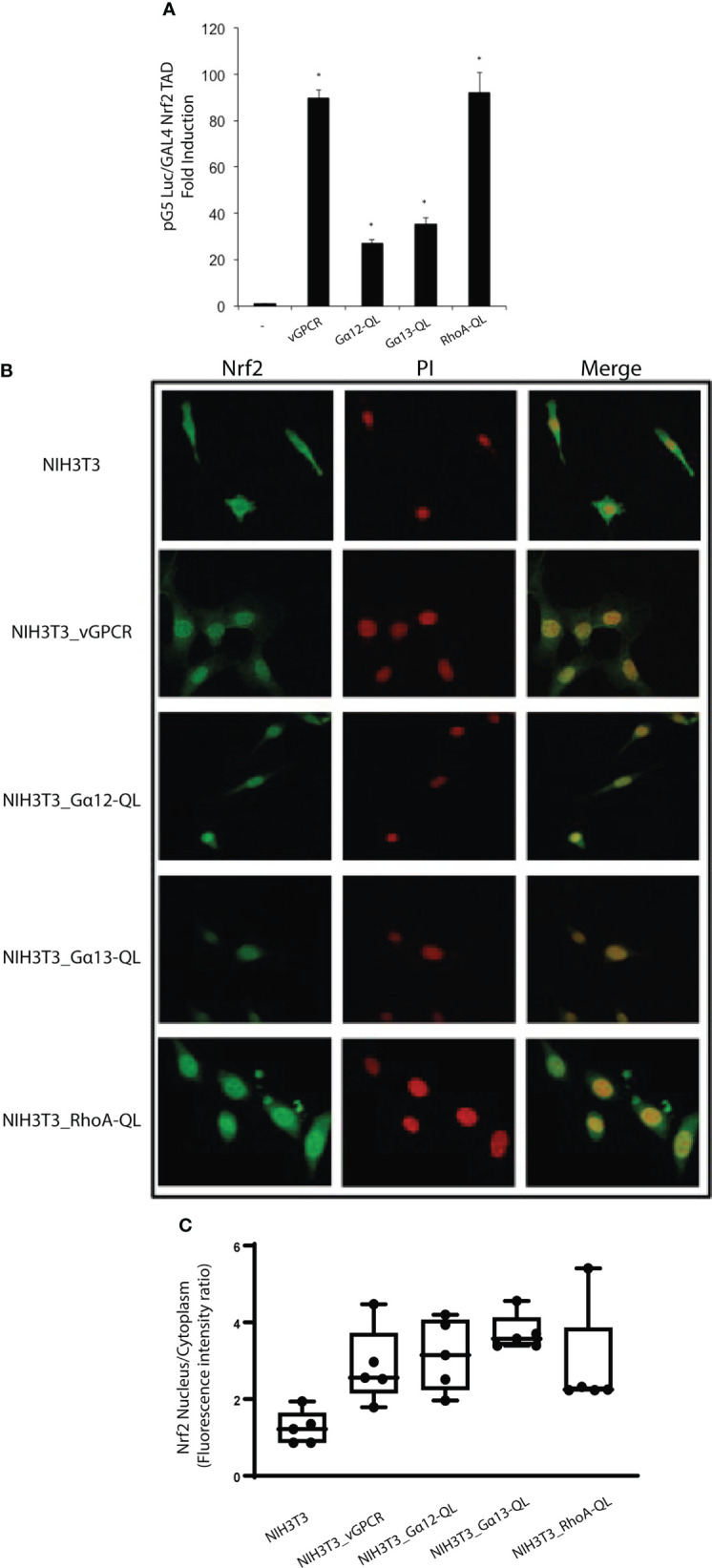
**(A)** Luciferase activity of the GAL4 reporter system to study transcriptional activation domains of transcription factors. pG5Luc and pGAL4_Nrf2TAD reporter plasmids were co-transfected with plasmids expressing vGPCR, Gα12-QL, Gα13-QL, and RhoA-QL. The results are expressed as fold induction relative to control cells (transfected with the reporter and an empty vector). **(B)** vGPCR, Gα12-QL, Gα13-QL, and RhoA-QL induced nuclear translocation of Nrf2. Confocal microscopy of NIH3T3 stable cell lines for the expression of vGPCR, Gα12-QL, Gα13-QL, and RhoA-QL were incubated with anti-Nrf2 and anti-rabbit FITC. For visualizing the nucleus, propidium iodide was used. Magnification 40X. **(C)** Quantification of fluorescence intensity for the nucleus/cytoplasm localization of Nrf2 in the different conditions from **(B)**. *p-value <*0.05 (*).

Like many transcription factors, Nrf2 has to be activated on its Transcriptional Activated Domain (TAD) to recruit the transcriptional machinery needed to activate the transcription of target genes. To determine if vGPCR affects TAD activation, we performed luciferase assays. We co-expressed a Gal4DBD–Nrf2TAD fusion protein and a Gal4 Binding Element upstream luciferase and evaluated reporter activity in control cells and cells overexpressing vGPCR, Gα12-QL, Gα13-QL, or RhoAQL. As shown in **Figure 3A**, vGPCR and the downstream signaling components can activate this promoter, suggesting that Nrf2-TAD targets vGCPR-triggered signaling. In basal conditions, Nrf2 localizes in the cytoplasm, and Keap1 drives it to proteasomal degradation, whereas in stimulated conditions, the complex Nrf2-Keap1 is dissociated, and Nrf2 translocated to the nucleus. To determine if vGPCR affects Nrf2 nuclear translocation, we performed immunofluorescence assays using a specific Nrf2 antibody on NIH3T3, NIH3T3_vGPCR, NIH3T3_Gα12-QL, NIH3T3_Ga13-QL and NIH3T3_RhoA-QL (all stable cell lines). As shown in **Figure 3B**, in NIH3T3 cells, Nrf2 has a predominantly cytoplasmic localization whereas, in the four stable cell lines that express vGPCR, Gα12-QL, Gα13-QL, or RhoA-QL, Nrf2 is found predominantly in the nucleus (**Figures 3B–C**). These results suggest that vGPCR and its effectors downstream have the same effect on Nrf2 nuclear translocation.

### vGPCR increases expression levels and phosphorylation of Nrf2

**Figure 4:**
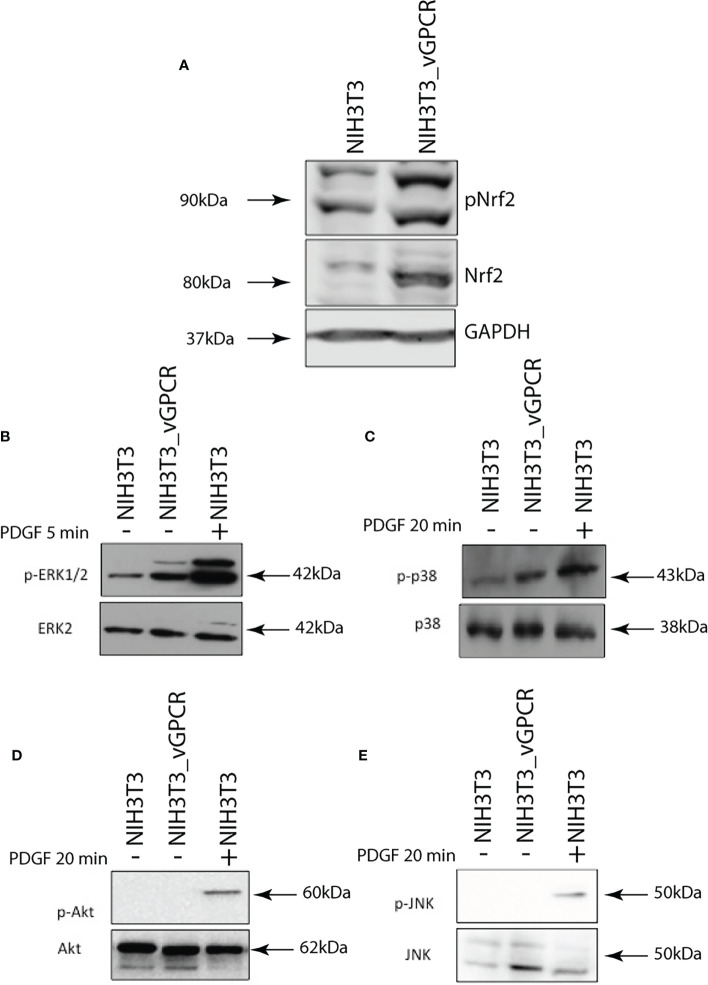
**(A)** Western Blot Assays performed in NIH3T3 and NIH3T3_vGPCR were evaluated for Nrf2 levels and Nrf2 phosphorylation using anti-phospho Nrf2 and anti- Nrf2. As loading control we used GAPDH. **(B)** Western Blot Assays performed in lysates of NIH3T3 and NIH3T3_vGPCR cells were evaluated for activation of ERK using anti-phospho ERK1/2 and anti-ERK2; as a control, we used NIH3T3 cells treated with PDGF 5 minutes. **(C)** Activation p38 using anti-phospho p38 and anti-p38; as control, we used NIH3T3 cells treated with Anisomycin for 20 minutes. **(D)** Activation of AKT using anti-phospho AKT and anti-AKT as a control, we used NIH3T3 cells treated with PDGF for 20 minutes. **(E)** Activation of JNK using anti-phospho JNK and anti-JNK as a control, we used NIH3T3 cells treated with PDGF for 20 minutes.

Given that it has been reported that Nrf2 is directed to proteasomal degradation and that in-stimulated conditions, it can be phosphorylated; we wondered if overexpression of vGPCR could stabilize the protein levels of Nrf2 and affect its phosphorylation levels. For this purpose, we performed Western Blots using lysates of NIH3T3 and NIH3T3_vGPCR cells and evaluated the levels of total and phosphorylated Nrf2 proteins in both cell lines. As seen in **Figure 4A**, vGPCR augments the level of total and phosphorylated Nrf2. These results suggest that vGPCR not only stabilizes Nrf2, but it also induces Nrf2 phosphorylation levels.

It is known that different post-translational modifications such as phosphorylation, ubiquitination, sumoylation, and many others can modify Nrf2 and Keap1. We evaluated different kinase activation pathways in our model to determine if phosphorylation on the Nrf2-Keap1 complex is a mediator of the vGPCR effect. We performed Western Blot assays with specific antibody pairs (phospho-protein and total protein) for four different relevant kinases, ERK1/2 (**Figure 4B**), p38 (**Figure 4C**), AKT (**Figure 4D**), and JNK (**Figure 4E**). In all cases, we evaluated NIH3T3, NIH3T3_vGPCR, and an appropriate positive control (see **Figures 4B–E**). As seen in **Figure 4**, vGPCR can activate ERK1/2 and p38 but not AKT and JNK in our model.

### vGPCR effects on Nrf2 transcriptional activity are mediated by ERK1/2

**Figure 5:**
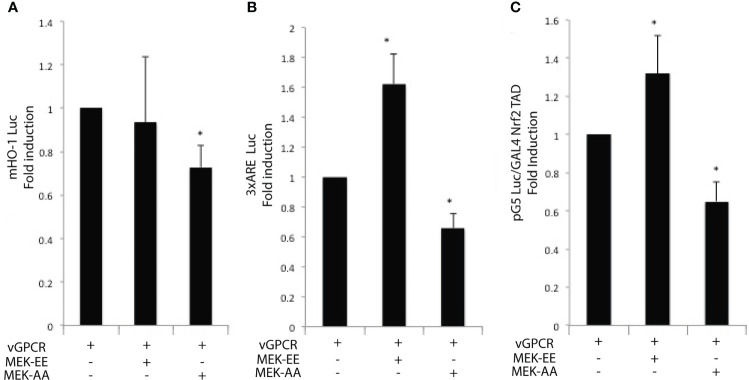
Luciferase activity of the murine HO-1 promoter **(A)**; 3xARE Luc **(B)** and the GAL4 – Nrf2 TAD reporter system **(C)** of NIH3T3 cells after transfection of vGPCR alone (bar one in each set); co-transfection of vGPCR and MEK-EE (bar 2 in each set) and co-transfection of vGPCR and MEK-AA (bar three in each set). *P-value <*0.05 (*).

As we showed, vGPCR affects Nrf2 transcriptional activity, so we wanted to know if the ERK1/2 pathway mediated this effect. For this aim, we performed Luciferase assays with the murine HO-1 promoter (**Figure 5A**), the 3xARE Luc (**Figure 5B**), and the GAL4–Nrf2TAD reporter system (**Figure 5C**). We transfected NIH3T3_vGPCR cells with the reporter construct. We activated the ERK1/2 pathway co- transfecting with expression plasmids for MEK-EE (constitutive activated) or inhibited the pathway co-transfecting with MEK-AA (dominant negative). **Figure 5A** shows that co-transfection of vGPCR and MEK-EE did not produce a higher effect in HO-1 promoter activation compared to vGPCR alone. This might be due to a saturation of the system when activation of the signaling axis to this particular reporter construct is already ignited by vGPCR. On the other hand, co-transfection of vGPCR with MEK-AA diminished the effect observed with vGPCR alone. **Figures 5B, C** showed a higher effect when co-transfecting vGPCR with MEK-EE than vGCPR alone. According to this result, co-transfection with vGPCR and MEK-AA produced a decrease in reporter activation concerning vGPCR alone. These results suggest that the ERK1/2 pathway mediates the vGCPR effect on Nrf2 transcriptional activity, which might have participated in HO-1 promoter activation.

### vGPCR effect on Nrf2 nuclear translocation is mediated by ERK1/2

**Figure 6:**
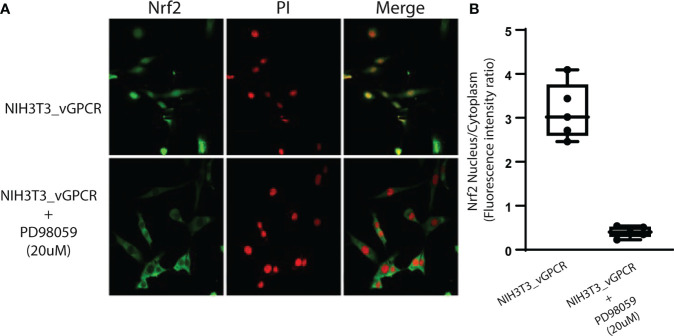
Inhibition of ERK1/2 activity block Nrf2 nuclear translocation. **(A)** Confocal microscopy of NIH3T3 stable cell lines for vGPCR control or treated with PD98059 20uM and incubated with anti-Nrf2 and anti-rabbit FITC. For visualizing the nucleus, propidium iodide was used. Magnification 40X. **(B)** Quantification of fluorescence intensity for the nucleus/cytoplasm localization of Nrf2 in the different conditions from **(A)**.

To evaluate the ability of vGPCR to induce Nrf2 nuclear translocation by ERK1/2 signaling, we performed immunofluorescence assays using an Nrf2-specific antibody treating NIH3T3_ vGPCR cells with or without the MEK inhibitor PD98059 (20uM) for 2hs. **Figure 6** shows that the inhibition of the ERK1/2 pathway by PD98059 impairs the nuclear translocation of Nrf2. The ratio of Nuclear Fluorescence/Cytoplasm Fluorescence drops from 2.5 in NIH3T3_vGCPR cells, which indicates nuclear localization to 0.5, indicative of cytoplasmic localization. This result suggests that the effect of vGPCR on Nrf2 nuclear localization is mediated by ERK1/2. Whereas vGPCR increases Nrf2 stability and phosphorylation and considering that vGCPR activates the ERK1/2 pathway, we wanted to test if the ERK1/2 pathway has a role in Nrf2 phosphorylation. As seen in **Supplementary Figure 2**, the inhibition of the ERK1/2 pathway did not produce a decrease in the phosphorylation levels of Nrf2 induced by vGPCR, suggesting that the effect of ERK1/2 may not be through direct phosphorylation of Nrf2 (**Supplementary Figure 2**).

### Silencing of Nrf2 impairs vGPCR-induced tumorigenesis in mice

**Figure 7:**
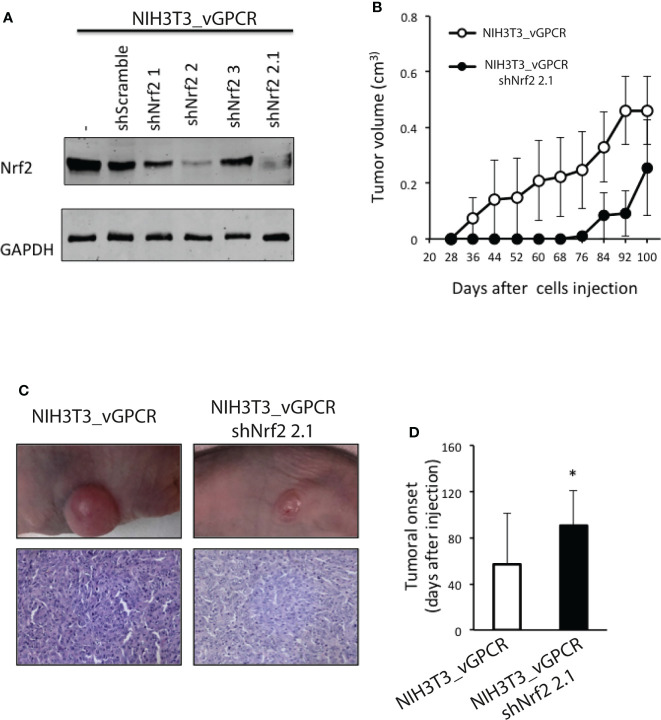
**(A)** Western Blot Assays performed in NIH3T3_vGPCR or different NIH3T3_vGPCR stable cell lines for the expression of shRNAs targeting Nrf2. We used an anti-Nrf2 antibody to detect Nrf2 levels in the different cell lines. **(B)** 1 X 10^6^ NIH3T3_vGPCR (control) or NIH3T3_vGPCR_shNrf2 2.1 cells were injected subcutaneously in the right flank of six and five nude mice respectively. Data are mean ± S.E.M expressed as tumor volume (cm^3^), calculated as Materials and Methods described. (Two-way repeated measures ANOVA, treatment factor F_1, 81_ = 2.47 p = 0.15). **(C)** An example of tumor-bearing mice from each group is depicted. H&E staining from representative tumors from mice injected with NIH3T3_vGPCR or NIH3T3_vGPCR_shNrf2 cells, respectively. **(D)** The tumor onset was defined as the first day of appearance of a measurable tumor. Data are mean ± S.E.M. NIH3T3_vGPCR: 57± 44.33; NIH3T3 _vGPCR_shNrf2: 90.6 ± 30.56. *p-value <*0.05 (*).

Whereas parental NIH3T3 cells are non-transformed, they acquire the capability to form foci in cell culture models and to induce tumors in nude mice when transfected by a plasmid construct expressing an oncogene. Thus, vGPCR-overexpressing NIH3T3 cells, but not the parental cells, have been reported to induce tumors when injected into nude mice (57). Prompted by our findings, we used these models to investigate whether silencing Nrf2 could affect vGPCR-induced tumorigenesis *in vivo*. For this aim, we transfected the NIH3T3_vGPCR cell line with different shRNAs directed against Nrf2 (NIH3T3_vGCPR–shNrf2) and with a control shRNA (NIH3T3_vGPCR–shScramble). We generated stable cell lines and evaluated them for Nrf2 expression levels. As shown in **Figure 7A**, stable cell lines named NIH3T3_vGPCR-shNrf2-2 and NIH3T3_vGPCR-shNrf2-2.1 showed lower levels of Nrf2 expression when compared with NIH3T3_vGPCR. We injected 1x10^6^ NIH3T3_vGPCR (control) or NIH3T3_vGPCR–shNrf2-2.1 cells into the right flank of six and five nude mice, respectively, and observed the mice twice a week. In concordance with the *in vitro* described effects upon HO-1 expression, silencing Nrf2 by shRNA strongly impacts the tumorigenic behavior of transformed cells *in vivo*. Tumors produced by NIH3T3_vGPCR–shNrf2_2.1 tend to be smaller than the ones observed in the NIH3T3_vGPCR group (**Figures 7B–C**); probably due to the significant delay in tumor onset found for Nrf2 silenced cells (**Figure 7D**).

### Nrf2 subcellular localization and activation by vGPCR in full KSHV genome bearing cells and KS-like mouse tumors

**Figure 8:**
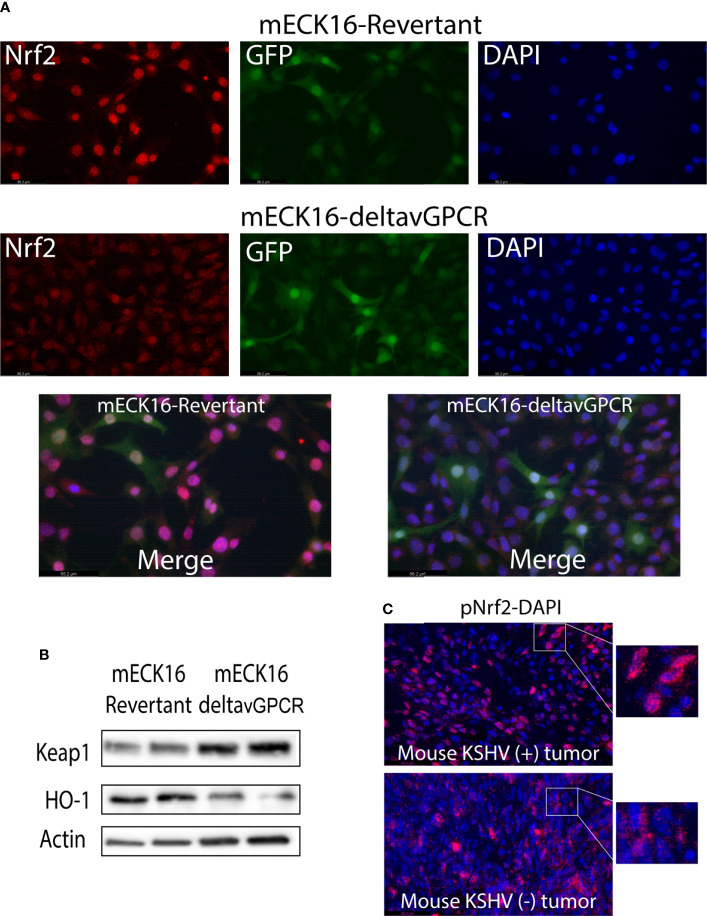
**(A)** Immunofluorescence analysis of mECK16-revertant and mECK16-ΔvGPCR cells to evaluate Nrf2 expression (red), GFP signal comes from the BAC16 plasmid (green), and nuclei were counterstained with DAPI (blue). **(B)** Western Blot Assays were performed in mECK16-revertant and mECK16-ΔvGPCR cell lines for the expression of Keap1 and HO-1; Actin was used as a loading control. **(C)** Immunofluorescence analysis of mouse KSHV (+) and mouse KSHV (–) tumors to evaluate Nrf2 phosphorylation (red), nuclei were counterstained with DAPI (blue).

To determine the specific contribution of vGPCR signaling to the subcellular localization of Nrf2 in the context of full KSHV genome-bearing cells, we used a previously described Bac16-delta vGPCR mutant or its revertant in mECK36 cells that have lost the Bac36 episome by lack of antibiotic selection, mECK16-deltavGPCR, and mECK16-revertant, respectively (53). In **Figure 8A**, top panel, Nrf2 subcellular localization is mainly nuclear in cells infected with the complete KSHV genome (mECK16-revertant cells), but in cells infected with a KSHV virus lacking vGPCR (mECK16-deltavGPCR cells) the Nrf2 subcellular localization shift to a more cytoplasmic signal (**Figure 8A** bottom panel). Moreover, western blot analysis of these cells showed that the cytoplasmic subcellular localization of Nrf2 in vGPCR mutant cells correlates with increased expression of Keap1 and decreased expression of HO-1, **Figure 8B**. Finally, we were able to show that in KSHV-positive [KSHV (+)] mouse KS-like tumors, Nrf2 activation (Nrf2 phosphorylation) and subcellular localization is mainly nuclear in contrast with that observed in KSHV-negative [KSHV (–)] mouse tumors, **Figure 8C**.

## Discussion

**Figure 9:**
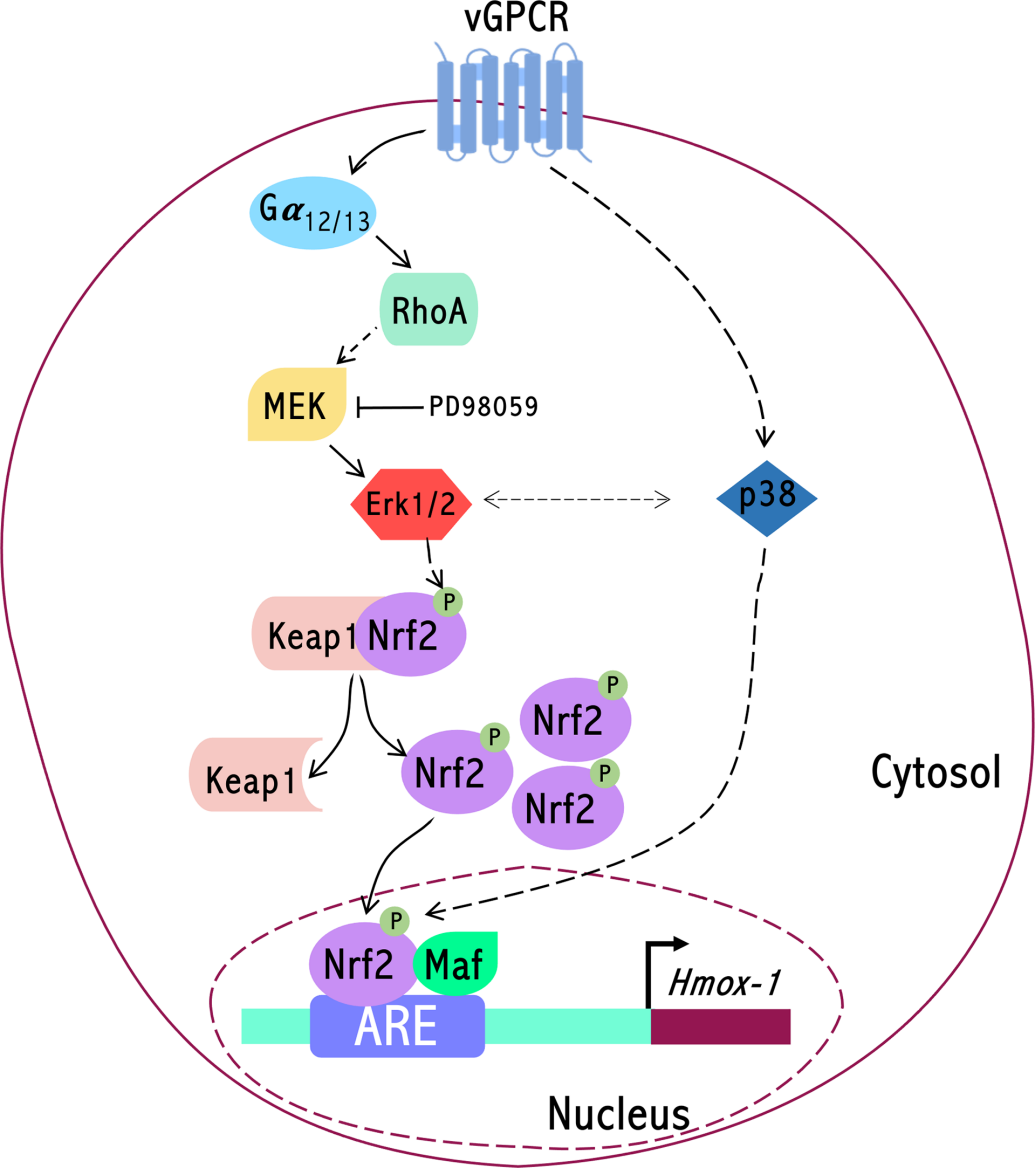
Signaling axis connecting the KSHV encoded oncogene vGPCR to the HO-1 promoter (Hmox-1) involves Gα12/13 and RhoA dependent Erk1/2 MAPK mediated activation of the transcription factor Nrf2.

Kaposi’s sarcoma (KS) is the most common tumor in AIDS patients, and the highly vascularized patient’s skin lesions are composed of the cells that derive from the endothelial tissue transformed by the KSHV virus (58). In previous works from our laboratory and collaborators, we have contributed to showing how the expression of vGPCR in fibroblasts produces transformation with foci formation in Petri dishes and tumors in nude mice. The lesions produced by these tumors in animals are rich in vessel irrigation, which resembles those of human patients with KS (31, 57). In addition, we have demonstrated that vGPCR induces the gene coding for HO-1 in both fibroblasts and endothelial cells and that this increase in HO-1 expression levels correlates with and is necessary for increased proliferation and cell survival. On the other hand, we have shown that the inhibition of HO-1 expression or its activity causes a reduction in the size of the vGPCR- induced tumors in mice (40). In cells transformed by vGPCR, it has been reported the activation of at least three major signal transduction pathways MAPKS ERK1/2, JNK, and p38 (59, 60). In addition, the cascade of PI3K/Akt has been reported as activated by the viral oncogene vGPCR. Activation of Akt would be dependent on PI3K and would have beta and gamma subunits of G proteins as intermediates (61). Small GTP binding proteins of the Rho family, such as RhoA and Rac1, as well as alpha subunits of heterotrimeric G proteins, increase their GTP loading in cells expressing vGPCR with their subsequent activation (40, 45). We have shown that vGPCR induces HO-1 expression and cell transformation using a pathway that sequentially includes the Gα12/13 and RhoA proteins (40, 45) **Figure 9**.

Regarding the role of HO-1 in the development of tumors, some results suggest that HO-1 can act as a cytoprotective enzyme, reducing the risk of developing some types of tumors. However, HO-1 is given a “dual” role. On one hand, it acts as a protective agent in healthy tissues, but on the other hand, it can act as an antiapoptotic and proangiogenic mediator. In one way or another, although the role of HO-1 as a target of vGPCR is clear, we still do not entirely know the mechanism that regulates its promoter expression. The transcription factors that bind to the HO-1 promoter are still poorly characterized as the signal transduction pathways that regulate them. The transcriptional regulation at the ARE site level is mainly controlled by Nrf2, a transcription factor of the leucine zipper-type (6) **Figure 9**.

According to our previous results and current global knowledge, we hypothesize that Nrf2 acts as a key factor in regulating the promoter of HO-1 by vGPCR. We contribute new data that links vGPCR with the promoter of HO-1, highlighting the fundamental role of Nrf2 as a mediator not only in the regulation of HO-1 but also in the tumorigenesis induced by vGPCR. We have described that the loss of the ARE element (located at the -4 Kb position) from the HO-1 promoter results in a decrease in reporter activity, indicating that this site is important in vGPCR mediated activation of HO-1 (**Figure 1B**).

We have determined the relevance of ARE sites on vGPCR-mediated activation. On one hand, we show that the deletion of a promoter region containing this sequence produces a decrease in the activation of the HO-1 promoter (**Figure 1B**) and, on the other hand, that vGPCR can activate a minimal promoter with ARE sites in tandem (3xARE Luc) (**Figures 1C–D**). Regarding the downstream elements of vGPCR, it was described by our group and co-workers, that vGPCR activates the HO-1 promoter through the small Gα12/13 G protein and RhoA (40, 45). In the present report, we demonstrated that both vGPCR and Gα12/13 activate ARE sites through RhoA and that they are not independent pathways since when co-transfected with a dominant negative form of RhoA, the effect produced by vGPCR and Gα12/13 was hampered (**Figures 1F–G**). This suggests that these elements are key in the activation of Nrf2. Once this was demonstrated, we considered it important to study the role of the transcription factor protein Nrf2. **Figures 1**, **2** show that Nrf2 is key for activating the HO-1 promoter and the ARE sites. We showed that Nrf2 can recruit the transcriptional machinery and induce expression of a reporter gene in response to vGPCR and downstream elements.

vGPCR activates different transcription factors that control different genes. In our model, although vGPCR can induce NF-κB through Rac1, and the HO-1 promoter has binding sites for this transcription factor, we provide evidence that Nrf2 has a key role as a transcription factor in the regulation of HO-1 mediated by vGPCR. Nrf2 activation seems to proceed *via* RhoA and not Rac1, although a more thorough study is currently being followed to determine the nature of Rac1 involvement. This suggests a fine regulation by a complex network of proteins that are differentially activated in different models and regulate gene expression orchestrating the biological response (proliferation, angiogenesis).

Another key aspect is the subcellular localization of Nrf2. It must be translocated to the nucleus to act as a transcription factor. We demonstrate here that both vGPCR and downstream elements can induce nuclear translocation of Nrf2 (**Figure 3**). This suggests that vGPCR is not only capable of inducing translocation to the nucleus of Nrf2 but that it may bind ARE elements and recruit the transcriptional machinery necessary for transcription initiation.

Work from different groups shows that vGPCR can activate different signaling pathways in different models. For example, Bais et al. have demonstrated that vGPCR activates the JNK and p38 pathways (57). In endothelial cells, vGPCR activates multiple pathways, including AMPc and AKT (41, 61, 62). The activation of this pathway has also been described in NIH3T3 cells. In this report, even using the same cell line, we have not seen AKT activation (**Figure 4D**), but we confirm that vGPCR activates the ERK1/2 and p38 pathways, as shown in **Figures 4B–C**. Numerous studies suggest that phosphorylation of Nrf2 may contribute to its regulation. Nrf2 contains serines, threonines, and tyrosines that can provide phosphorylation sites for various kinases. For example, it has been shown that PKC can phosphorylate Nrf2 in serine 40 (Neh2) and disrupt the association between Nrf2/Keap1, thus promoting the translocation of Nrf2 to the nucleus (63). Keum et al. demonstrated that p38 could phosphorylate Nrf2, promote its association with Keap1 and thus prevent its nuclear translocation (64). We have shown that vGPCR stabilizes and increases the phosphorylation levels of Nrf2 (**Figure 4A**).

Furthermore, vGPCR activates the ERK1/2 pathway and influences Nrf2 transcriptional activation (**Figure 5**) and nuclear translocation (**Figure 6**). However, we observed no effect on Nrf2 phosphorylation when we treated NIH3T3_vGPCR cells with the MEK inhibitor PD98059 (**Supplementary Figure 2**). This could be due, for example, to the fact that Nrf2 is being phosphorylated in several residues and that inhibition of the ERK pathway affects only certain amino acids, among which Serine 40 is not found to recognize the antibody used herein. Another possibility that justifies this result would be that vGPCR activates the phosphorylation of Nrf2 by an ERK-independent pathway and that ERK is regulating some proteins related to the import of Nrf2 to the nucleus. In this way, the inhibition of the ERK pathway would impede the translocation to the nucleus of Nrf2 in an indirect fashion. We cannot determine with the techniques performed whether the increase of Nrf2 is due to an increase in the half-life of Nrf2 or if it is due to an increase in the expression of the Nrf2 gene. However, our results clearly show the involvement of ERK1/2 as an intermediary between the vGPCR-Gα12/13-RhoA axis and nuclear translocation of Nrf2, as well as its transcriptional activation.

By performing experiments in nude mice, we show that the tumorigenic effect of vGPCR is affected by the silencing of Nrf2 (**Figure 7**). Cells that clearly show a decrease in Nrf2 expression by Western Blot were able to form tumors but with a markedly significant delay. Interestingly, the immunohistochemical analysis of control and experimental groups has shown detectable Nrf2 levels. However, experimental groups have shown only residual expression levels. This data tells us about a positive selection of cells expressing Nrf2 produced *in vivo* on the population of injected cells and provides extra data regarding the importance of expressing Nrf2 so that these cells can develop a tumor.

Studies published by Gjyshi et al. have shown that *de novo* infection of endothelial cells by KSHV leads to an increase in Nrf2 expression, an increase in the nuclear fraction of Nrf2, and an increase in phosphorylation levels of Nrf2 (46, 47). In addition, they have demonstrated that the increase in Nrf2 stability is not due directly to the dissociation of Nrf2 from Keap1 but also increases the expression of Nrf2 and, consequently, HO-1. It is noteworthy that those works used the complete genome of KSHV so that different proteins may be involved in regulating HO-1. To test the relevant oncogenic role of vGPCR in Nrf2 activation in the context of the complete KSHV genome, we used a deletion mutant of vGPCR and showed the lack of vGPCR induced an increase in cytoplasmic localization of Nrf2 correlated with a downregulation of HO-1 expression. Moreover, when we compare mouse KS-like KSHV (positive) tumors with KSHV (negative) tumors, we also show an increase in phospho-Nrf2 nuclear localization in KSHV (positive) tumors (**Figure 8**).

Many of the experiments in this study are performed using NIH3T3 cells as a biological scenario. These cells are widely used for validating oncogene activity, allowing consistency with our previous signaling studies (40, 45). Anyway, to test an environment more related to cells infected with KSHV, we have performed experiments that show vGPCR-dependent control of HO-1 expression under the control of Nrf2, with a model developed in Dr. Mesri’s laboratory using mouse bone marrow cells of the endothelial cell lineage expressing the complete KSHV genome in its wild type form or variants.

It is also important to note that many pathways begin to interact when infected with the complete genome of KSHV. For example, regarding the increase of HO-1, we mention that KSHV increases the stability and phosphorylation of Nrf2, but it is also known that the BACH1 (repressor of HO-1 expression) mRNA is negatively regulated by viral miRNA miR-K12-11 (65, 66). This might imply that the expression of vGPCR and miR-K12-11 are two independent mechanisms that converge on the increase of HO-1.

We have previously shown that vGPCR is one of the key genes for tumor development induced by infection with KSHV (56). Our laboratory had previously reported that vGPCR targeted the HO-1 promoter through the Gα12/13-RhoA proteins and that the development of these tumors was mediated by HO-1; we have also demonstrated that pharmacological inhibition or decreased HO-1 expression produced a decrease in tumor size (40). Throughout this work, we have been able to deepen the study on the effects of vGPCR on the expression of HO-1. We have shown that vGPCR not only activates Gα12/13-RhoA, but these proteins are signaling towards ARE sites present in the HO-1 promoter and that the transcription factor Nrf2 is key in this regulation. We have also shown that vGPCR-Gα12/13-RhoA signal to MAPK ERK1/2 and that vGPCR activates p38 MAPK. Regarding the role of ERK, we have demonstrated that it affects translocation to the nucleus of Nrf2 and the transcriptional activation of its transactivation domain. Finally, experiments in nude mice show that the tumorigenic effect of vGPCR is affected by the silencing of Nrf2 since mice injected with NIH3T3_vGPCR-shNrf2 cells showed a delay in tumor development. Altogether, our results show that vGPCR signals through Gα12/13, RhoA, and Erk1/2 to the HO-1 promoter in an Nrf2-dependent manner, as sketched in **Figure 9**. Our report points out Nrf2 and its associated factors as a putative pharmacological target for controlling cell growth in cells transformed by KSHV oncogenes providing the basis to focus our efforts in considering Nrf2 and associated proteins as therapeutic targets in KS treatment.

## Data availability statement

The original contributions presented in the study are included in the article/**Supplementary Material**. Further inquiries can be directed to the corresponding author.

## Ethics statement

All animal studies were carried out according to Institutional Animal Care and Use Committee of the Facultad de Ciencias Exactas y Naturales (FCEN) University of Buenos Aires approved protocol and local government regulations (Servicio Nacional de Sanidad y Calidad Agroalimentaria, RS617/2002, Argentina).

## Author contributions

SD: Conceptualization, data curation, formal analysis, methodology, validation, visualization, investigation, writing-original draft; RA: Conceptualization, data curation, formal analysis, methodology, validation, investigation; MV: Conceptualization, data curation, formal analysis, methodology, validation, investigation; NJ: Conceptualization, data curation, formal analysis, methodology, validation, investigation, writing-original draft, writing-review, and editing; ME: Conceptualization, investigation, supervision, funding acquisition, project administration; CO: Conceptualization, data curation, methodology, investigation, writing-original draft, writing-review and editing, supervision, funding acquisition, project administration. All authors contributed to the article and approved the submitted version.

## Funding

This work was supported by the NIH grants CA136387 (to ME) and CA221208 (to ME and CO); by the Florida Biomedical Foundation, Bankhead Coley Foundation grant 3BB05 (to ME), by Ubacyt Grant 20020150100200BA (to CO), Ubacyt Grant Proyecto # 01/W949.20020100100949 (to CO) and by National Agency of Scientific and Technological Promotion: PICT 2015-3436 (to CO).

## Acknowledgments

We are thankful to Dr. Maria Julia Marinissen and Dr. Tamara Beatriz Tanos for insightful discussions that fueled the early stages of the work presented in this manuscript.

## In memoriam

Dr. Enrique Mesri, inspiring artist and teacher, outstanding investigator, sadly passed away days before acceptance of this manuscript. We are indebted to him for his guidance, his energetic commitment to work, his willingness to share enthusiasm and remarkable scientific contributions.

## Conflict of interest

The authors declare that the research was conducted in the absence of any commercial or financial relationships that could be construed as a potential conflict of interest.

## Publisher’s note

All claims expressed in this article are solely those of the authors and do not necessarily represent those of their affiliated organizations, or those of the publisher, the editors and the reviewers. Any product that may be evaluated in this article, or claim that may be made by its manufacturer, is not guaranteed or endorsed by the publisher.
