# Supplementary figures and images for: A major role for Nrf2 transcription factors in cell transformation by KSHV encoded oncogenes
Source: Front Oncol. 2022 Sep 21;12:890825. doi: 10.3389/fonc.2022.890825 (PMC9534600; doi:10.3389/fonc.2022.890825)

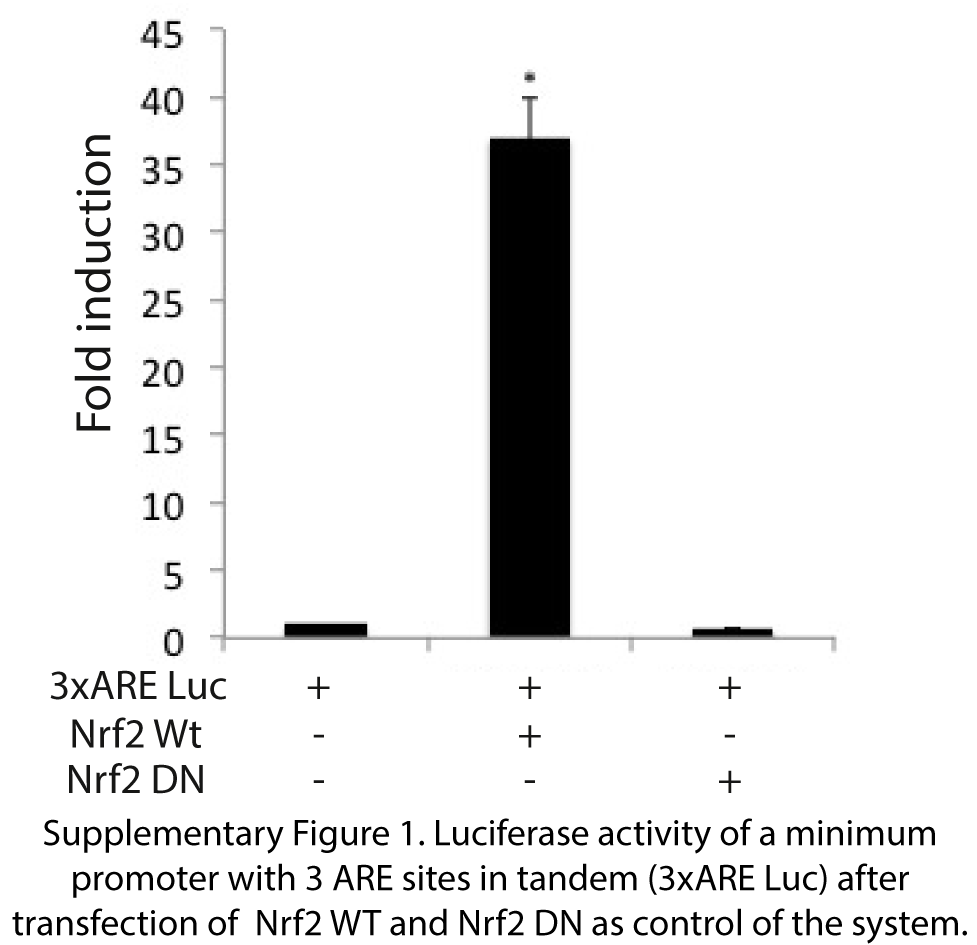

Supplement: Supplementary file 1 [file Image_1.tif]

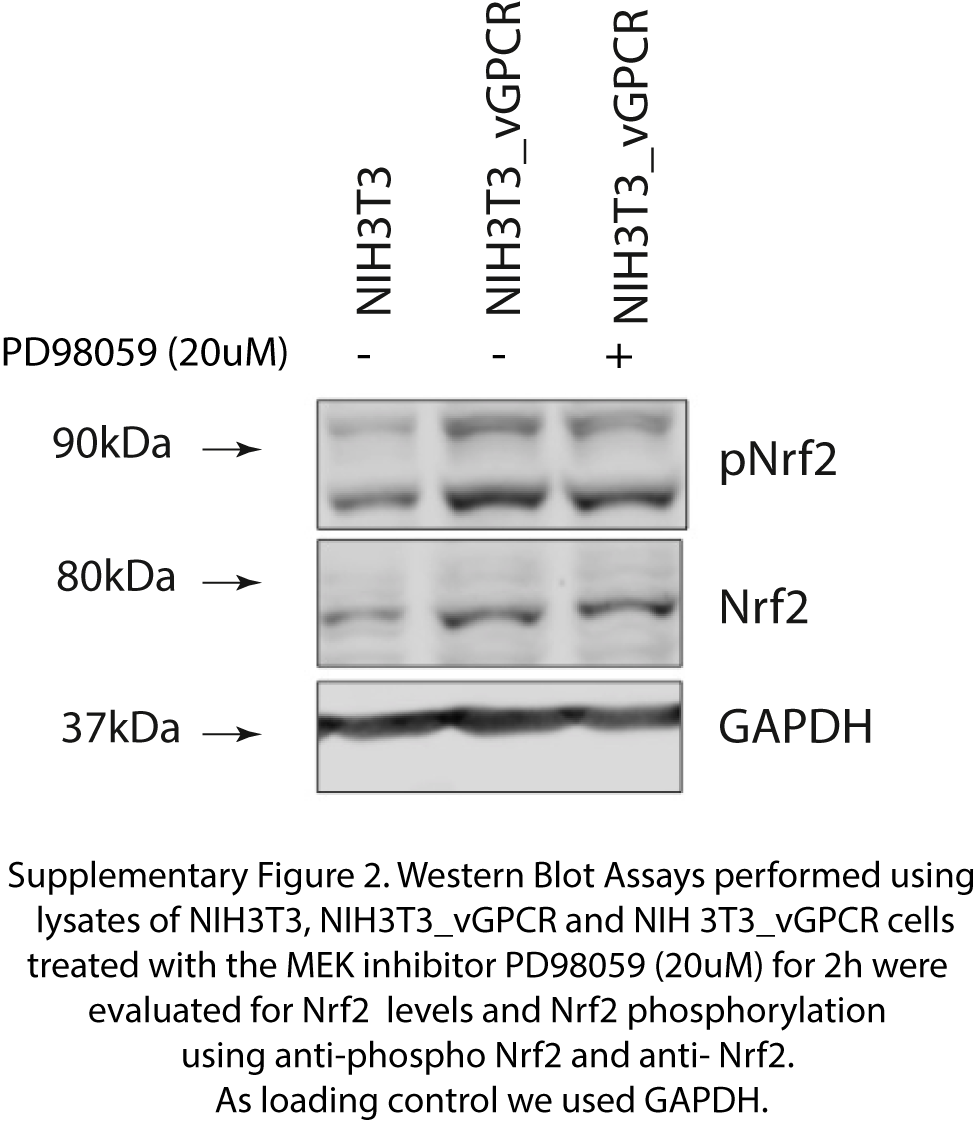

Supplement: Supplementary file 2 [file Image_2.tif]
